# Semantic Neighborhood Effects for Abstract versus Concrete Words

**DOI:** 10.3389/fpsyg.2016.01034

**Published:** 2016-07-06

**Authors:** Ashley N. Danguecan, Lori Buchanan

**Affiliations:** Department of Psychology, University of Windsor, WindsorON, Canada

**Keywords:** visual word recognition, semantic neighborhood density, concrete words, abstract words, lexical decision, progressive demasking

## Abstract

Studies show that semantic effects may be task-specific, and thus, that semantic representations are flexible and dynamic. Such findings are critical to the development of a comprehensive theory of semantic processing in visual word recognition, which should arguably account for how semantic effects may vary by task. It has been suggested that semantic effects are more directly examined using tasks that explicitly require meaning processing relative to those for which meaning processing is not necessary (e.g., lexical decision task). The purpose of the present study was to chart the processing of concrete versus abstract words in the context of a global co-occurrence variable, semantic neighborhood density (SND), by comparing word recognition response times (RTs) across four tasks varying in explicit semantic demands: standard lexical decision task (with non-pronounceable non-words), go/no-go lexical decision task (with pronounceable non-words), progressive demasking task, and sentence relatedness task. The same experimental stimulus set was used across experiments and consisted of 44 concrete and 44 abstract words, with half of these being low SND, and half being high SND. In this way, concreteness and SND were manipulated in a factorial design using a number of visual word recognition tasks. A consistent RT pattern emerged across tasks, in which SND effects were found for abstract (but not necessarily concrete) words. Ultimately, these findings highlight the importance of studying interactive effects in word recognition, and suggest that linguistic associative information is particularly important for abstract words.

## Introduction

Answers to the question of how meaning (semantics) is derived from printed words advance our knowledge of basic reading processes, and provides insight into the storage and retrieval of semantic knowledge. As a field we are working toward a fully comprehensive theory of semantic processing, and the goal of the present study is to contribute to this effort. Specifically, we examined the results of four experiments that compared recognition response time (RT) patterns (across tasks varying in explicit semantic demands) for concrete and abstract words. Importantly, we examined these two word types within the context of a list that also included a linguistic semantic variable, known as semantic neighborhood density (SND; Durda and Buchanan, 2008). The data from these experiments frame several proposals regarding how a comprehensive semantic theory may address distinctions between concrete versus abstract word representations.

By definition, concreteness is a measure of the extent to which a word’s referent can be experienced by the senses (Dove, 2015). In this way, a broad distinction can be made between two word types: concrete and abstract. Concrete words typically refer to concepts that are spatially circumscribed and physically tangible (e.g., *TABLE, KITCHEN, BASKETBALL*), whereas abstract words (e.g., *BRAVERY, FULFILLMENT, ACADEMIA*) often refer to concepts consisting of social, event-related, or introspective information (Barsalou and Wiemer-Hastings, 2005; Borghi and Cimatti, 2009). As expressed by Barsalou (2008, p. 634), “Because the scientific study of concepts has primarily focused on concrete concepts, we actually know remarkably little about abstract concepts, even from the perspective of traditional cognitive theories”. Indeed, as noted by Recchia and Jones (2012) most models of word recognition were developed on data derived from studies using concrete word stimuli, and the applicability of these models to abstract word processing has yet to be fully established. Arguably, the domains of experience expressed by abstract words (e.g., social information, introspective states) may not be adequately captured by concrete words. Therefore, one of the objectives of the present study is to contribute to our knowledge of abstract words.

Importantly, concrete and abstract words appear to be represented in different ways in the mental lexicon as evidenced by performance differences. For example, many studies have found that concrete words are both recognized and recalled more easily than are abstract words, a phenomenon known as the concreteness effect (reviewed e.g., Paivio, 1991; Schwanenflugel, 1991). Several theories addressing representational distinctions between concrete versus abstract words have been developed based on evidence from cognitive and neuropsychological investigations. As **Table 1** shows, despite extensive examinations of differences between abstract and concrete words, there remains no consensus regarding the nature of these processing differences, and the mechanisms responsible for them. A meaningful discussion of the strengths and limitations of each theory is beyond the scope of this paper. However, a general overview of these theories is provided here to illustrate how others have conceptualized the concrete/abstract distinction. For example, certain theories propose that concrete concepts are more semantically complex than abstract ones (i.e., dual-coding theory; Paivio, 1971; context availability theory; Schwanenflugel and Shoben, 1983), whereas others suggest that abstract concepts are more semantically complex (e.g., perceptual symbol systems; Barsalou, 1999). Moreover, various theories propose different ideas for what characterizes the nature of concrete versus abstract concepts, though there has been little discussion regarding how these explanations may be integrated. For example, it has been proposed that the semantic content of concrete versus abstract words varies by type of sensorimotor experience (perceptual symbol systems theory; Barsalou, 1999), type of semantic relationship with other concrete and abstract words (qualitatively different representational hypothesis; Crutch and Warrington, 2005), or proportion of embodied versus linguistic information (theory of embodied abstract semantics; Vigliocco et al., 2009). The current investigation seeks to contribute to this body of literature by exploring concrete and abstract word recognition across a range of tasks within the context of another semantic variable, SND, which is a language-based semantic variable that captures richness information for both word types.

**Table 1:** Summary of concrete versus abstract word processing models, with their basic tenets, predictions, and supporting research.

Theory	Basic tenets	Predictions regarding concrete versus abstract word processing	Empirical support for predictions
Dual Coding Theory (Paivio, 1971)	• Concrete words are represented by linguistic and imagistic codes; abstract words are only represented by a linguistic code.	• Concrete words should be processed faster than abstract words.	Reviewed e.g., Paivio (1991)

Context Availability Theory (Schwanenflugel and Shoben, 1983)	• Concrete words are associated with stronger and denser associations to contextual information compared to abstract words.	• Concrete words should be processed faster when presented in isolation. • There should be no difference between concrete and abstract word RTs when context is provided.	Reviewed, e.g., Schwanenflugel (1991)

Qualitatively Different Representational Hypothesis (Crutch and Warrington, 2005)	• Concrete words are primarily organized by semantic similarity (i.e., same category, similar features), whereas abstract words are primarily organized by semantic association (i.e., shared linguistic context or ‘real life’ associations).	• When processing concrete words, similarity-based connections are identified faster than association-based connections • When processing abstract words, association-based connections are identified faster than similarity-based connections	Crutch et al. (2009)

Perceptual Symbol Systems (Barsalou, 1999)	• Both concrete and abstract word processing involves simulation of sensorimotor experiences (i.e., perceptual symbols) associated with a given concept. • Concrete and abstract words differ in the content of these simulations. Introspective, social, and event knowledge is central to abstract simulations, and object knowledge is central to concrete simulations.	• Human generated properties for concrete and abstract concepts will vary in content. • Concrete words should elicit primarily object-related properties, while abstract words should elicit introspective, social, and event-related properties	Barsalou and Wiemer-Hastings (2005) Wiemer-Hastings and Xu (2005)

Hub-and-Spoke Model (Rogers et al., 2004; Lambon Ralph et al., 2007; Patterson et al., 2007)	• The anterior temporal lobes bilaterally serve as a central amodal hub for semantic knowledge by integrating knowledge from amodal cortical areas	• Damage to the anterior temporal lobes should impair knowledge for both concrete and abstract words	Pobric et al. (2007, 2009), Hoffman and Lambon Ralph (2011)

Theory of Embodied Abstract Semantics (Vigliocco et al., 2009)	• Both concrete and abstract words are composed of embodied/experiential (sensorimotor, affective) and linguistic associative information. Concrete words are primarily composed of sensorimotor information. Abstract words are primarily composed of emotional and linguistic information.	• When concrete and abstract words are controlled for sensorimotor information, there should be an advantage for abstract words. Affective associations should account for this abstract word advantage.	Kousta et al. (2011)

Broadly speaking, studies on how semantics influence the word recognition process have focused on how various object-based and language-based variables impact RTs on a variety of tasks (reviewed, e.g., Pexman, 2012). Object-based models (e.g., feature-based models) classify related words in terms of the physical similarity of their referents, and thus, they easily lend themselves to studies involving concrete words. Conversely, according to language-based models, the semantic richness of a word may be measured according to the number of contexts in which the word appears (Adelman et al., 2006), the number of human-generated distinct first associates (Nelson et al., 1998), or the number of unrelated meanings (ambiguity; Rodd et al., 2002). Words may also vary in the distinctiveness of the contexts in which they appear (i.e., contextual/semantic diversity), and Jones et al. (2012) describe how lexical strength may develop as a function of word use in varied contexts. A related variable that is central to the present study is semantic neighborhood size, whereby words with many neighbors are those that often appear with many other words in linguistic corpora. The number of these different co-occurrences is captured in a word’s semantic neighborhood size that may be considered related to semantic richness (e.g., Buchanan et al., 2001). Moreover, the distribution of these neighbors may differ such that the average number of near neighbors (i.e., semantic neighbors clustered closely around the target word in semantic space) may also vary. This variation in distribution of semantic neighbors refers to a word’s SND, (Durda and Buchanan, 2008).

Semantic neighborhood density refers to the average proximity of semantic neighbors to a target word as defined by a global co-occurrence model (WINDSORS; Durda and Buchanan, 2008). Thus, SND is a linguistically derived variable that is meant to serve as a measure of the overall distribution of neighbors within a given word’s semantic space. In this way, semantic neighborhoods may be described as relatively sparse (i.e., low SND) or clustered (i.e., high SND). SND was first studied in the context of reading performance in individuals with deep dyslexia (Buchanan et al., 1996). The effects of SND on a neurologically intact sample were first studied by Buchanan et al. (2001) using the term “semantic distance”, which referred to the average distance between a target word and its 10 closest neighbors as defined by a global co-occurrence model (HAL; Lund and Burgess, 1996). More specifically, it was assumed that words with high semantic distance should have a sparse neighborhood since the 10 closest neighbors would be relatively distant from the target^1^ On the other hand, words with low semantic distance should have a dense semantic neighborhood since the 10 closest neighbors would be relatively close to the target word. According to hierarchical regression analyses, semantic distance accounted for unique variance in lexical decision RTs even after accounting for previously established lexico-semantic variables (i.e., log frequency, orthographic neighborhood size, word length, imageability). Buchanan et al.’s (2001) results suggest that word recognition is facilitated by having a large and dense semantic neighborhood (relative to a small and sparse semantic neighborhood). These findings were replicated in the context of a go/no-go semantic categorization task requiring participants to make animal/non-animal judgments (Siakaluk et al., 2003). Such results are consistent with the idea of semantic feedback models, which propose that words with rich semantic representations provide strong feedback to lexical-level orthography, thus facilitating visual word recognition (Hino and Lupker, 1996; Pexman et al., 2002; Yap et al., 2012, 2015). Specifically, if lexical (word/non-word) decisions are primarily based on orthography (i.e., does this look like a word?), then having a richer semantic representation (i.e., low semantic distance) should facilitate responding by providing strong top-down feedback from semantics to orthography.

More recently, Mirman and Magnuson (2008) explored how attractor dynamics could contribute to an understanding of SND facilitation effects. These authors independently manipulated the effects of near versus distant neighbors and analyzed RTs from a semantic categorization task. The results revealed slower RTs for words with many near neighbors relative to words with few near neighbors (i.e., many distant neighbors). The authors attributed this effect to the former having greater competition effects from very semantically similar words. From an attractor dynamics framework, distant neighbors are thought to create a gravitational gradient that speeds settling to the correct “attractor” (i.e., target word), thereby facilitating recognition RTs. On the other hand, near neighbors are believed to create conflicting sub-basins that slow settling to the correct attractor, which slows recognition RTs by increasing the likelihood of near neighbor competition. In an attempt to test this attractor dynamics hypothesis, Mirman and Magnuson (2008) analyzed settling patterns and model RTs for the words in the above experiment using a computational semantic model trained by O’Connor et al. (2006) to activate semantic features. Consistent with their behavioral data, their model produced results reflecting inhibitory effects of near neighbors. Importantly, however, these data do not directly contribute to an understanding of SND (as previously described) because the words modeled in the computational model were derived from feature-based norms (McRae et al., 2005). Nonetheless, given the interdependence of feature-based and language-based semantics discussed above, the potential effects of neighborhood distribution on recognition RTs should also be investigated using global co-occurrence norms. Recent work in this area using the WINDSORS global co-occurrence definition of SND (Macdonald, 2013; Danguecan and Buchanan, 2014, unpublished) found support for the idea that words with many near neighbors are processed more slowly than words with few near neighbors in both lexical decision and semantic categorization tasks. Although the present study uses the WINDSORS model to study semantic neighborhood effects (Durda and Buchanan, 2008), other distributional models such as Hyperspace Analog to Language (HAL; Lund and Burgess, 1996), Correlated Occurrence Analog to Lexical Semantics (COALS; Rohde et al., 2004), Latent Semantic Analysis (LSA; Landauer and Dumais, 1997), Bound Encoding of the AGgregate Language Environment (BEAGLE; Jones and Mewhort, 2007), OrBEAGLE (Kachergis et al., 2011), Random Permutation Model (Sahlgren et al., 2008), the Topic model (Griffiths et al., 2007), and HiDEx (Shaoul and Westbury, 2010) have also contributed extensively to our knowledge of semantic phenomena.

Pertaining to the present study, we argue that SND, a distributional, language-based measure of semantics, is particularly useful for studying both concrete and abstract words because SND is able to provide information about both word types (McRae and Jones, 2013). Object-based models, because of their focus on physical attributes, are arguably less able to capture abstract word semantics. However, some have asserted that distributional variables such as SND are not grounded in perception because semantic relations are solely based on the associations between words (i.e., symbol grounding problem; Glenberg, 1997; Glenberg and Robertson, 2000; French and Labiouse, 2002). In response to this criticism, Durda et al. (2009) demonstrated that WINDSORS (the model from which SND is derived) is also capable of generating perceptual features. Therefore, it could be argued that SND is at least partially grounded, and that abstract words are indirectly grounded through their linguistic relationships with other concrete (grounded) concepts (Recchia and Jones, 2012). For example, the abstract words *FLIGHT* and *ACADEMIA* are associated with other concrete (grounded) concepts such as *AIRPLANE* and *PROFESSOR*, respectively.

The argument that semantic representations are not static cognitive entities has become increasingly popular in the psycholinguistic literature, as evidenced by recent investigations on the task-specific effects of various semantic variables (e.g., Pexman et al., 2008; Yap et al., 2012; Zdrazilova and Pexman, 2013). RTs from any single visual word recognition task reflect time devoted to semantic processing, as well as other task-specific requirements/strategies (Balota and Yap, 2006). Indeed, it is assumed that there are no process-pure measures of visual word recognition or semantic processing. In light of this, a potentially useful approach is to compare how the effects of semantic variables are impacted by various task demands, which Balota and Yap (2006) termed the *task-appropriate processing framework*. Basically, this approach assumes that distinct lexico-semantic processes are central to various language-processing tasks. For example, in a naming task for which participants are instructed to read words aloud, the pathway between phonology (how a word sounds) and orthography (how a word looks) is emphasized. This may be contrasted with the visual lexical decision task in which participants must distinguish between printed letter strings that are meaningful real words or meaningless non-words. In this case, the pathway between orthography and semantics is emphasized. Below, we argue that the task-appropriate processing framework is also useful for studying the effects of semantic variables across tasks.

Pexman et al. (2007) proposed that tasks emphasizing explicit semantic processing may be better at capturing abstract word semantics as compared to tasks that do not emphasize explicit semantics (e.g., lexical decision task). Specifically, these authors compared levels of cortical activation between concrete and abstract words using fMRI during an explicit semantic task (i.e., semantic categorization: decide if the word represents a food/beverage). Abstract words produced more extensive cortical activation than concrete words, and this was attributed to the ability of the explicit semantic task to fully activate abstract word representations. Based on research in embodied cognition by Barsalou and Wiemer-Hastings (2005), Pexman et al. (2007) concluded that abstract words may be more complex/rich than concrete words. In light of decades of work proposing that concrete words are more richly represented than abstract words (e.g., Shallice and Warrington, 1975; Paivio, 1991; Schwanenflugel, 1991; Adorni and Proverbio, 2012), this is a relatively novel and intriguing argument.

## The Present Study

The argument that abstract semantic effects are better captured by explicit semantic tasks requires a systematic comparison of concrete and abstract words across tasks varying in explicit semantic demands. This is the objective of the present study. Specifically, word recognition RT patterns for the same stimulus set (words varying in concreteness and SND) were compared across four tasks varying in semantic engagement. To start, we conducted two different lexical decision tasks: In Experiment 1 we used a standard lexical decision task with non-pronounceable non-words, and in Experiment 2 we used a go/no lexical decision task with pronounceable non-words. Participants should be able to primarily rely on orthography to distinguish between non-pronounceable non-words and real words. In comparison, when participants must distinguish between meaningful and meaningless (but pronounceable) letter strings, they are encouraged to access semantics to make a lexical decision (Coltheart et al., 1977; Binder et al., 2003). Therefore, Experiment 2 is presumably more reliant on explicit semantic access than Experiment 1. In Experiment 3 we used the progressive demasking task (Grainger and Segui, 1990), in which a stimulus word is rapidly interspersed with a masking stimulus (e.g., “#####”), and participants perceived the stimulus word as gradually emerging from the mask. In a previous study (Dunabeitia et al., 2008), this task uncovered semantic effects without explicit meaning judgment, similar to the lexical decision task. However, unlike the lexical decision task, the PDT requires unique word identification. Since the PDT is meant to slow down unique visual word identification, this task may serve to uncover additional semantic effects that may be masked by the other tasks in this study. Finally, to examine explicit semantic effects, we developed another novel task, known as the sentence relatedness task. In other psycholinguistic studies, the semantic categorization task was used as an explicit semantic task (Forster and Shen, 1996; Hino et al., 2002; Siakaluk et al., 2003; Pexman et al., 2007). However, the decision categories in these studies have often required the use of control words that are concrete (e.g., is the word is a food/beverage?; does the word represent a living or non-living entity?). This results in participants viewing more concrete than abstract words overall. The sentence relatedness task was developed in an attempt to resolve this issue. Specifically, participants were instructed to decide whether a target word was related to a previously presented sentence or not. In summary, the present study employed a wide range of semantic demands for the purpose of charting the potential flexibility of concreteness and SND effects.

## Methods and Procedures

### Operational Definitions

#### Concreteness

Although words theoretically vary along a concreteness continuum (ranging from very concrete to very abstract), the existence of two distinct groups (i.e., concrete and abstract) is supported by the bimodal distribution of data from studies on human concreteness ratings, in which each mode is centered in each half of the concreteness scale (Nelson and Schreiber, 1992; Wiemer-Hastings et al., 2001). Therefore, for the purposes of the present study, stimulus words were categorized as being concrete or abstract. Within the potential pool of low and high SND words, potential stimulus words were categorized by the authors as being either concrete or abstract. Specifically, a word was labeled as “concrete” if it referred to a physically tangible entity, and a word was labeled as “abstract” if it referred to a non-physically tangible entity.

#### Semantic Neighborhood Density

**FIGURE 1:**
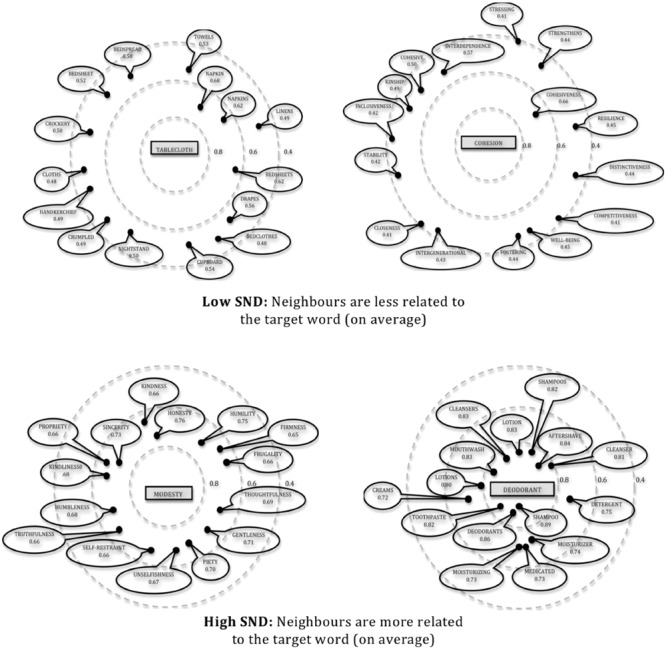
**Two-dimensional theoretical representations of low versus high SND words with their closest 15 neighbors**.

In accordance with previous investigations of SND conducted by Macdonald (2013) and Danguecan and Buchanan (2014, unpublished), SND is defined in the current study as the average degree of similarity between a target stimulus word and all other words in its semantic neighborhood (as derived from a global co-occurrence model) using a cut-off of 3.5 SDs (WINDSORS; Durda and Buchanan, 2008). Therefore, SND is meant to serve as an index of the distribution of neighbors within a given word’s semantic space. Using hierarchical regression analyses, Macdonald (2013) demonstrated that using a standard score cutoff of 3.5 SDs best predicted lexical decision RT data from the Balota et al. (1999) corpus. SND values range from 0 to 1^2^, but to allow for factorial manipulation of SND within a stimulus set, words were categorized as being either low SND or high SND. Low and high SND words were selected from the bottom and top 33% of the words within the WINDSORS database, respectively. Low SND words (SND values equal to or less than 0.347) are those with smaller SND values (i.e., closer to 0) and have weakly related neighbors that are relatively distant. On the other hand, high SND words (SND values equal to or greater than 0.375) are those with higher SND values (i.e., closer to 1) and have closely related neighbors that are tightly clustered. See **Figure 1** for a simplified illustration of low versus high SND representations. Importantly, low and high SND words were controlled for semantic neighborhood size and therefore had the same approximate number of neighbors, but the *distribution* of their semantic neighbors was manipulated.

### Stimulus Development

The same experimental words were used for all experiments. The critical stimulus set is composed of 44 concrete and 44 abstract common nouns. Half of the abstract words and half of the concrete words are low SND and half are high SND. The words are matched across conditions (i.e., concrete-low SND, concrete-high SND, abstract-low SND, abstract-high SND) on the following lexical/semantic variables as measured by WINDSORS (Durda and Buchanan, 2008): word length, frequency, number of syllables, and semantic neighborhood size. All words have an orthographic neighborhood size of 0, 1, or 2, with an overall average of 0.26. All of the words are low frequency (i.e., fewer than 10 per million). The difference between the mean SND values of the low and high SND conditions is statistically significant (*p* < 0.05), whereas the difference between the mean SND values of the concrete and abstract words within the low and high SND conditions is not statistically significant (*p* > 0.05). A summary of the experimental word characteristics is provided in **Table 2**. The full stimulus set is presented in the Supplementary Materials. Additional items are described in the context of the relevant experiments below.

**Table 2:** Means and standard deviations for word length, number of syllables, frequency (Freq), orthographic neighborhood size (ON), semantic neighborhood size (SN), and semantic neighborhood density (SND) per experimental word condition.

Word type	Length	#Syllables	Freq	ON	SN	SND
***Concrete***						
Low SND	8.41 (1.14)	3.05 (0.65)	1.24 (1.29)	0.40 (0.67)	212.55 (39.43)	0.34 (0.01)
High SND	8.41 (1.14)	3.05 (0.65)	1.26 (1.32)	0.05 (0.21)	217.86 (40.83)	0.39 (0.02)
***Abstract***						
Low SND	8.41 (1.14)	3.05 (0.65)	1.43 (1.01)	0.37 (0.65)	210.77 (41.90)	0.34 (0.01)
High SND	8.41 (1.14)	3.05 (0.65)	1.38 (1.29)	0.18 (0.39)	214.91 (38.07)	0.38 (0.01)

### General Procedures for All Experiments

#### Participant Recruitment and Inclusion Criteria

Following Research Ethics Board approval, University of Windsor undergraduate students were recruited through the Psychology Participant Pool, and provided their written informed consent prior to participation. Separate samples of participants were recruited for each experiment, and they received partial course credit upon completion of their respective task. All participants were required to be at least 18 years of age, report having learned English as a first language, and report normal or corrected-to-normal vision.

#### Task Software and Display Details

All tasks were administered on a Dell PC using the Windows 7 operating system. Direct RT (Version 2012.4.0.166; Empirisoft Corporation; New York, NY, USA) was used to administer the lexical decision task (with non-pronounceable non-words), go/no-go lexical decision task, and sentence relatedness task. For these experiments, words were presented in the middle of the screen in size 24, bold-faced font. Dedicated software was used for the progressive demasking task due to the especially precise timing requirements for stimulus presentations (Dufau et al., 2008), as further explained below.

#### Task Administration

To ensure proper understanding of task instructions, participants completed a series of practice trials supervised by a research assistant prior to each experiment. Accuracy feedback was provided on all practice trials. For all experiments, trials were presented in random order.

### Experiment 1: Lexical Decision Task (with Non-pronounceable Non-words)

Participants viewed each experimental word or non-pronounceable letter string one at a time. They were instructed to indicate with a key press (as quickly and as accurately as possible) whether the letter string formed a real English word or a non-word. Pronouceable non-words (generated using an in-house program) were matched to the experimental words on letter length and orthographic neighborhood size. The first vowel was then replaced with a consonant to make the non-words non-pronounceable.

### Experiment 2: Go/No-Go Lexical Decision Task

Participants viewed each experimental word or pronounceable letter string one at a time. They were instructed to press a key (as quickly and as accurately as possible) when presented with a real word. No action was required if presented with a non-word, and they waited 2500 ms for the next trial to begin. In addition to the experimental words, the original set of non-words produced for Experiment 2 (before they were made non-pronounceable) was used for Experiment 3.

### Experiment 3: Progressive Demasking Task

Each trial of the PDT consisted of an experimental word-mask pair with a fixed combined duration of 233 ms. The masking stimulus was a series of 10 hash marks (##########), corresponding with the length of the longest experimental words. Within each trial, the ratio of the word-mask pair increased whereby the experimental word was initially presented for 1 display cycle (14 ms), and the mask was presented for the remainder of the trial (219 ms). As each trial progressed, the word presentation duration increased by one cycle each time (i.e., 28, 42, 56…ms), while the mask duration decreased by the same proportion (i.e., 205, 191, 177…ms). This resulted in the participants perceiving each word as “emerging” from the mask. They were instructed to press the spacebar as soon as they were able to read the word. The stimulus word disappeared once the spacebar was pressed, at which point they were prompted to type the word they just read. Participants’ typed responses were manually checked for accuracy so that only correct RTs were statistically analyzed. Responses provided after 3262 ms were excluded as the words were clearly presented without the masking stimulus at this point. Given that this task does not require control words, only the experimental words were used as stimuli.

### Experiment 4: Sentence Relatedness Task

For this task participants were presented with a short sentence, which remained on the screen for as long as needed for comprehension. They were then instructed to press the space bar, which prompted the presentation of a single (experimental or control) word. Participants were instructed to press the space bar (as quickly and as accurately as possible) if they believed the word was not related to the preceding sentence. They were instructed to do nothing if they believed the word was related to the preceding sentence, and the next trial began after 2500 ms. This way, all experimental words (corresponding to unrelated sentence-word pairs) should have produced a behavioral response, whereas the control words (corresponding to related sentence-word pairs) should have produced no response. To maximize consistency between the sentences, each was formulated using the same sentence structure. An example sentence that preceded the experimental target word *FREEZER* is “The child rolled the colored marbles on the ground”, whereas an example sentence that preceded the control word *BALLOON* is “The child popped the party decorations on the ground.” Note that the subject, prepositions, and ending words for the sentences are the same; only the verbs and nouns changed in their relatedness to their matched experimental or control word.

## Results

### Outlier Identification

The following procedure was used to identify outliers for all experiments. After removal of all incorrect responses, participants and stimulus items with less than 70% accuracy were excluded from subsequent statistical analyses. At this point outliers were excluded, which were defined as RTs deviating more than 2.5 SDs from the mean of a given word condition (i.e., concrete – low SND, concrete – high SND, abstract – low SND, abstract – high SND), after responses faster than 200 ms or slower than 3000 ms were excluded.

### General Statistical Procedures

First, incorrect responses, participants and stimulus items with insufficient (<70%) accuracy rates, and outliers were removed. Then mean RTs per condition were calculated for each participant to conduct the subject analysis (*F*_1_), and for each stimulus item to conduct the item analysis (*F*_2_). As such, for all experiments, concreteness and SND were considered within-subject variables in the subject analysis, and as between-item variables in the item analysis. RTs and error rates were analyzed separately.

For the subject analyses, mean RTs and error rates for each condition across participants were analyzed using a within-subjects analysis of variance (ANOVA). For the item analyses, mean RTs and error rates for each condition across stimulus items were analyzed using a between-items ANOVA. Planned contrasts (*t*-tests) were also conducted to compare low and high SND means within the concrete and abstract word groups (i.e., low versus high SND concrete words; low versus high SND abstract words).

For all experiments, refer to **Table 3** for samples sizes, demographic information (i.e., age and gender), number of participants and items excluded, as well as the percentage of observations removed due to error and the outlier analysis (described above). The results from each individual experiment will be described below. For subject RT comparisons across tasks, please refer to **Table 4**.

**Table 3:** Sample sizes (with number of females and males), mean participant age, number of participants and items excluded, and percentage of observations excluded for all experiments.

	Experiment 1: non-pronounceable non-word lexical decision task	Experiment 2: go/No-Go lexical decision task	Experiment 3: progressive demasking task	Experiment 4: sentence relatedness task
Final Sample Size	40 (34F, 6M)	41 (30F, 11M)	45 (^∗^gender info not available due to computer error)	41 (35F, 6M)
Mean Participant Age	21.33	21.49	(^∗^information not available due to computer error)	20.12
# Participants Excluded	0	0	2	1
# Items Excluded	1 Abstract-Low SND (fervor)	1 Abstract-High SND (*accolade*); 1 Concrete-Low SND (*bayonet*); 1 Abstract-Low SND (fervor)	1 Concrete-Low SND (*prairie*); 1 Concrete-High SND (*embroidery*); 1 Abstract-High SND (*sustenance*)	0
% Incorrect	9.50	9.32	9.38	9.17
% Outliers	2.14	3.29	2.46	3.10

**Table 4:** Subject mean RTs in milliseconds (with standard deviations), and subject mean error rates (with standard deviations) per condition for all experiments.

Word type	Experiment 1: non-pronounceable non-word lexical decision task (*n* = 40)		Experiment 2: go/No-Go lexical decision task (*n* = 41)		Experiment 3: progressive demasking task (*n* = 43)		Experiment 4: sentence relatedness task (*n* = 40)	
	Mean RT (msec)	Mean Error (%)	Mean RT (msec)	Mean Error (%)	Mean RT (msec)	Mean Error (%)	Mean RT (msec)	Mean Error (%)
CONCRETE Low SND	691.07 (92.92)	5.80 (6.59)	827.92 (126.69)	6.50 (6.96)	1670.19 (205.03)	8.31 (7.85)	891.57 (156.61)	6.93 (7.84)
CONCRETE High SND	704.44 (93.31)	6.70 (7.13)	840.21 (122.81)	5.88 (6.91)	1703.62 (232.34)	8.42 (9.17)	885.49 (146.15)	3.64 (5.47)
ABSTRACT Low SND	676.71 (95.43)	5.95 (6.26)	829.28 (114.74)	6.97 (8.32)	1784.25 (242.24)	7.51 (7.99)	952.42 (182.05)	6.59 (8.73)
ABSTRACT High SND	748.94 (114.06)	8.18 (7.15)	968.10 (148.86)	12.89 (12.24)	1855.83 (274.85)	15.84 (12.11)	993.91 (187.10)	6.82 (9.26)

### Experiment 1: Lexical Decision Task (with Non-pronounceable Non-words)

#### RT Analysis

**FIGURE 2:**
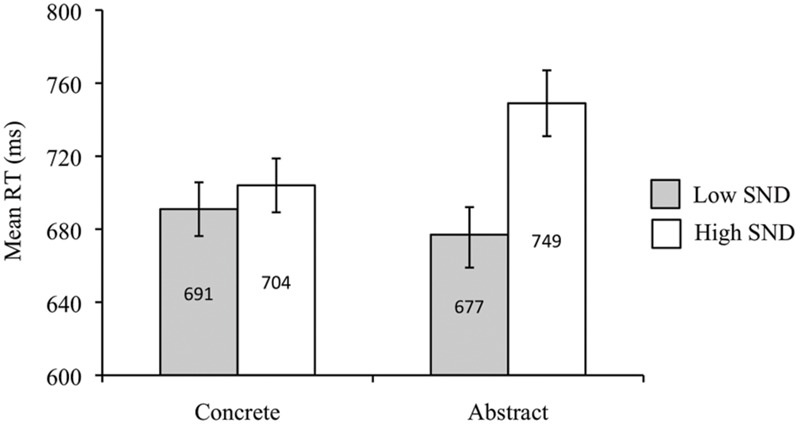
**Experiment 1 (lexical decision task with non-pronounceable non-words) subject analysis mean RTs.** Error bars represent standard error.

A main effect of concreteness was obtained in the subject analysis, such that concrete words produced faster RTs than abstract words [*F*_1_(1,39) = 4.82, *p* < 0.05, $\eta_{p}^{2}$ = 0.11], though this effect was not replicated in the item analysis [*F*_2_(1,83) = 1. 30, *p* = 0.26]. Both the subject and item analyses revealed faster RTs for low SND compared to high SND words [*F*_1_(1,39) = 64.62, *p* < 0.05, $\eta_{p}^{2}$ = 0.62; *F*_2_(1,83) = 11.01, *p* < 0.05, $\eta_{p}^{2}$ = 0.12]. There was also an interaction [*F*_1_(1,39) = 40.00, *p* < 0.05, $\eta_{p}^{2}$ = 0.51; *F*_2_(1,83) = 5.29, *p* < 0.05, $\eta_{p}^{2}$ = 0.06], whereby abstract – low SND words produced faster RTs than abstract – high SND words [*t*_1_(39) = -10.10, *p* < 0.05; *t*_2_(41) = -3.84, *p* < 0.05], though there was no effect of SND within the concrete word group [*t*_1_(39) = -1.91, *p* = 0.06; *t*_2_(42) = -0.74, *p* = 0.46]. Mean RTs from the subject analysis are presented in **Figure 2**.

#### Error Analysis

Analysis of mean error rates for subjects and items revealed no effect of concreteness [*F*_E1_(1,34) = 0.74, *p* = 0.40; *F*_E2_(1,56) = 0, *p* = 0.99]. Participants made more errors when responding to high SND compared to low SND words as indicated by the subject analysis [*F*_E1_(1,34) = 6.80, *p* < 0.05, $\eta_{p}^{2}$ = 0.17], though the effect was non-significant in the item analysis [*F*_E2_(1,56) = 0.004, *p* = 0.95]. Finally, the concreteness by SND interaction was non-significant [*F*_E1_(1,34) = 1.07, *p* = 0.31; *F*_E2_(1,56) = 2.46, *p* = 0.12].

### Experiment 2: Go/No-Go Lexical Decision Task

#### RT Analysis

**FIGURE 3:**
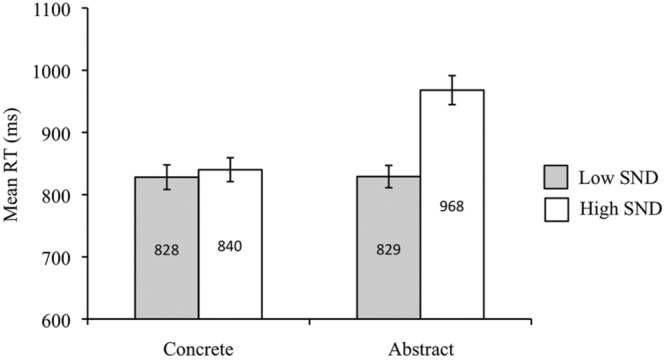
**Experiment 2 (go/no-go lexical decision task with pronounceable non-words) subject analysis mean RTs.** Error bars represent standard error.

Analysis of mean RTs revealed that participants responded more quickly to concrete words than to abstract words [*F*_1_(1,40) = 48.24, *p* < 0.05, $\eta_{p}^{2}$ = 0.55; *F*_2_(1,81) = 8.93, *p* < 0.05, $\eta_{p}^{2}$ = 0.10]. Faster RTs were also produced for low SND compared to high SND words [*F*_1_(1,40) = 91.77, *p* < 0.05, $\eta_{p}^{2}$ = 0.70; *F*_2_(1,81) = 12.37, *p* < 0.05, $\eta_{p}^{2}$ = 0.13]. Moreover, there was an interaction [*F*_1_(1,40) = 73.87, *p* < 0.05, $\eta_{p}^{2}$ = 0.65; *F*_2_(1,81) = 8.59, *p* < 0.05, $\eta_{p}^{2}$ = 0.10], whereby for abstract words, participants responded more quickly to low SND than to high SND words [*t*_1_(40) = -10.32, *p* < 0.05; *t*_2_(31.839^3^) = -4.30, *p* < 0.05], though no such effect of SND was evident for concrete words [*t*_1_(40) = -1.71, *p* = 0.10; *t*_2_(41) = -0.44, *p* = 0.66]. Mean RTs from the subject analysis are presented in **Figure 3**.

#### Error Analysis

Analysis of mean error rates per participant revealed a pattern consistent with the RT results summarized above. Participants committed more errors when presented with abstract words than concrete words [*F*_E1_(1,33) = 23.38, *p* < 0.05, $\eta_{p}^{2}$ = 0.42], with this effect approaching significance in the item analysis [*F*_E2_(1,43) = 3.60, *p* = 0.07, $\eta_{p}^{2}$ = 0.08]. There were also more errors made in response to high SND words than to low SND words [*F*_E1_(1,33) = 14.79, *p* < 0.05, $\eta_{p}^{2}$ = 0.31], though this effect was not replicated in the item analysis [*F*_E2_(1,43) = 1.04, *p* = 0.32, $\eta_{p}^{2}$ = 0.02]. The subject error analysis revealed a concreteness by SND interaction [*F*_E1_(1,33) = 22.33, *p* < 0.05, $\eta_{p}^{2}$ = 0.40], whereby there were more errors for abstract – high SND words than abstract – low SND words [*t*_E1_(33) = -5.01, *p* < 0.05], but no difference in errors between concrete – high SND and concrete – low SND words [*t*_E1_(33) = -0.30, *p* = 0.77]. However, the interaction term in the item analysis was non-significant [*F*_E2_(1,43) = 1.17, *p* = 0.29].

### Experiment 3: Progressive Demasking Task

#### RT Analysis

**FIGURE 4:**
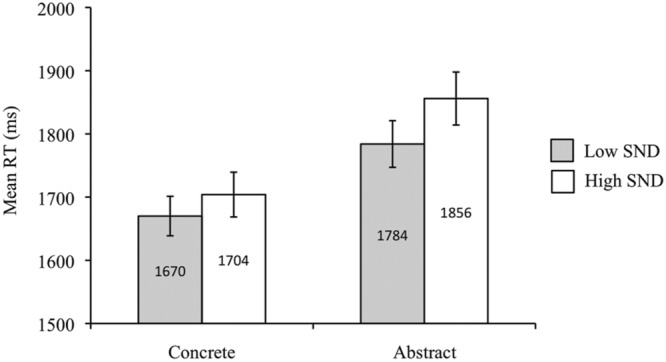
**Experiment 3 (progressive demasking task) subject analysis mean RTs.** Error bars represent standard error.

Overall, concrete words were recognized more quickly than abstract words [*F*_1_(1,42) = 81.14, *p* < 0.05, $\eta_{p}^{2}$ = 0.66; *F*_2_(1,81) = 13.46, *p* < 0.05, $\eta_{p}^{2}$ = 0.14]. The subject analysis revealed faster RTs for low SND words compared to high SND words [*F*_1_(1,42) = 22.86, *p* < 0.05, $\eta_{p}^{2}$ = 0.35], though this effect was non-significant in the item analysis [*F*_2_(1,81) = 1.92, *p* = 0.17, $\eta_{p}^{2}$ = 0.02]. There was also a concreteness by SND interaction in the subject analysis [*F*_1_(1,42) = 4.50, *p* < 0.05, $\eta_{p}^{2}$ = 0.10], whereby there was a larger effect of SND for abstract words [*t*_1_(42) = -4.88, *p* < 0.05] than for concrete words [*t*_1_(42) = -2.44, *p* < 0.05]; however, the interaction term was non-significant in the item analysis [*F*_2_(1,81) = 0.31, *p* = 0.58]. Mean RTs from the subject analysis are presented in **Figure 4**.

#### Error Analysis

Analysis of mean error rates revealed that participants did not commit more errors as a function of concreteness [*F*_E1_(1,37) = 0.99, *p* = 0.33; *F*_2_(1,54) = 0.86, *p* = 0.36]. Consistent with the slower observed RTs for high SND words, participants also made more errors in response to high SND words compared to low SND words [*F*_E1_(1,37) = 5.33, *p* < 0.05, $\eta_{p}^{2}$ = 0.13], though this was not observed in the item analysis [*F*_E2_(1,54) = 0.01, *p* = 0.93]. The concreteness by SND interaction term was non-significant in both the subject and item analyses [*F*_E1_(1,37) = 2.51, *p* = 0.12; *F*_E2_(1,54) = 0.36, *p* = 0.57].

### Experiment 4: Sentence Relatedness Task

#### RT Analysis

**FIGURE 5:**
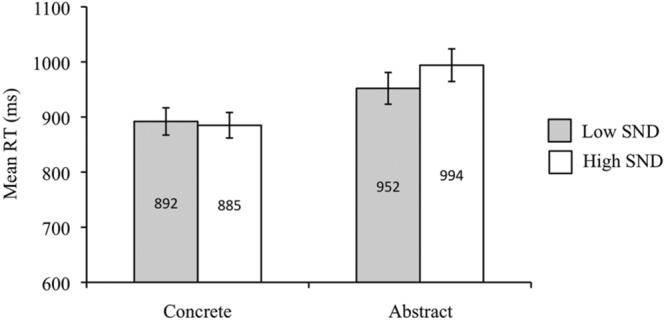
**Experiment 4 (sentence relatedness task) subject analysis mean RTs.** Error bars represent standard error.

Participants responded more quickly to concrete than abstract words [*F*_1_(1,39) = 84.26, *p* < 0.05, $\eta_{p}^{2}$ = 0.68; *F*_2_(1,84) = 31.14, *p* < 0.05, $\eta_{p}^{2}$ = 0.27]. RTs were faster for low SND compared to high SND words in the subject analysis [*F*_1_(1,39) = 5.04, *p* < 0.05, $\eta_{p}^{2}$ = 0.11], though this effect was non-significant in the item analysis [*F*_2_(1,84) = 1.45, *p* = 0.23]. The concreteness by SND interaction was also significant in the subject analysis [*F*_1_(1,39) = 7.92, *p* < 0.05, $\eta_{p}^{2}$ = 0.17] but not in the item analysis [*F*_2_(1,84) = 2.51, *p* = 0.12]. The subject analysis interaction revealed that for abstract words, low SND words had faster RTs than high SND words [*t*_1_(39) = -3.40, *p* < 0.05], though there was no effect of SND for concrete words [*t*_1_(39) = 0.56, *p* = 0.58]. Mean RTs from the subject analysis are presented in **Figure 5** below.

#### Error Analysis

Consistent with the finding that abstract words had slower RTs than concrete words, abstract words also produced higher error rates than concrete words overall in the subject analysis [*F*_E1_(1,28) = 6.65, *p* < 0.05, $\eta_{p}^{2}$ = 0.19] but not in the item analysis [*F*_E2_(1,41) = 0.65, *p* = 0.43]. There was no difference in error rates between low and high SND words [*F*_E1_(1,28) = 2.56, *p* = 0.12; *F*_E2_(1,41) = 0.17, *p* = 0.68). The subject analysis revealed a concreteness by SND interaction [*F*_E1_(1,28) = 7.12, *p* < 0.05, $\eta_{p}^{2}$ = 0.20], such that participants made more errors for concrete – low SND words than for concrete – high SND words [*t*_E1_(28) = 3.54, *p* < 0.05], though there was no such effect for abstract words [*t*_E1_(28) = -0.70, *p* = 0.49]. However, the interaction term was non-significant in the item analysis [*F*_E2_(1,41) = 3.00, *p* = 0.09].

### Linear Mixed Effects Analyses

The results from our four experiments demonstrate that concreteness and SND impact word recognition RTs on several tasks. Although our stimulus set is carefully controlled for a number of psycholinguistic variables known to influence recognition RTs (i.e., orthographic neighborhood size, frequency, length, number of syllables), it may be argued that our results may be confounded by lack of statistical control of other semantic variables that tend to differ between concrete and abstract words. Specifically, some propose that abstract words are more emotionally valenced than concrete words (Vigliocco et al., 2009; Kousta et al., 2011). Abstract words also tend to be learned later than concrete words, thus making subjective age of acquisition (AoA) ratings higher for abstract than concrete words (e.g., Baddeley et al., 1982; Frith, 1985). To examine the impact of valence and AoA on our results, we analyzed the data from all experiments using linear mixed effects analyses in R (R Development Core Team, 2011) using the lme4 package (Bates et al., 2015); *p*-values were obtained for the fixed effects using the lmerTest package (Kuznetsova et al., 2013). AoA ratings were retrieved from Kuperman et al. (2012)^4^ and we collected valence ratings from a separate sample of 45 University of Windsor undergraduate students (mean age = 20 years; 39 females, 6 males) using the same recruitment procedures described previously for Experiments 1 to 4^5^.

For each experiment, AoA, valence, concreteness, and SND were treated as fixed effects, whereas participants and items were treated as random effects. The results from these analyses are presented in **Table 5**. Most importantly, the data show at least a trend toward significance for the concreteness by SND interaction term in Experiments 1, 2, and 4^6^. Due to the relatively small number of items in each condition, the item analyses would attenuate any subject effects. However, consistent with the ANOVA results summarized above, the data continue to reveal significant (or close to significant) concreteness by SND interaction effects when AoA and valence are included in the analyses.

**Table 5:** Estimates for fixed effects parameters (along with *p*-values based on the *t*-statistic) for all experiments.

Predictor	Experiment 1: non-pronounceable non-word lexical decision task (*n* = 40)	Experiment 2: go/No-Go lexical decision task (*n* = 41)	Experiment 3: progressive demasking task (*n* = 43)	Experiment 4: sentence relatedness task (*n* = 40)
AoA	5.29^∗^	6.41^∗^	4.44^∗^	2.45^∗^
Valence	-0.78	-0.95	-1.31	0.66
Concreteness	4.15^∗^	4.33^∗^	1.25	-0.51
SND	3.73^∗^	4.17^∗^	0.75	1.76 (^∗^)
Conc. X SND	-3.24^∗^	-4.28^∗^	-1.05	-1.90 (^∗^)

*^∗^*p* < 0.05. (^∗^) indicates trend toward significance, *p* < 0.09.*

## Discussion

The main objective of this study was to chart the semantic effects of words varying in concreteness and SND by comparing word recognition RTs across a series of tasks ranging in semantic engagement. Specifically, we used tasks for which semantics was presumed to be useful but not necessary (Experiment 1: lexical decision task with non-pronounceable non-words; Experiment 2: go/no-go lexical decision task), a task for which word identification (but not explicit meaning judgment) was required (Experiment 3: Progressive Demasking Task), and a task for which explicit meaning processing was required (Experiment 4: sentence relatedness task). It has been suggested (Pexman et al., 2007; Yap et al., 2012) that semantic effects are more directly examined using tasks that explicitly require participants to process meaning compared to those for which explicit semantic engagement is not necessary (e.g., lexical decision task; Hino and Lupker, 1996). Across tasks, our data show that SND effects were consistently observed for abstract (but often not concrete) words, regardless of the depth of semantic processing required.

Interestingly, the pattern of RTs was the same for the Experiment 1 lexical decision task and the Experiment 4 sentence relatedness task, even though the sentence relatedness task presumably required much more explicit semantic processing than the lexical decision task. Concrete words consistently produced faster RTs than abstract words, a finding that is in keeping with most research comparing these two word types (reviewed, e.g., Paivio, 1991) and suggests that concrete word representations possess qualities that make them easier to process compared to abstract words. However, it is unlikely that this difference can be attributed to abstract words having relatively impoverished semantic representations. Across experiments there was also a significant interaction whereby abstract (but not concrete) words produced an effect of SND such that abstract-low SND words were recognized faster than abstract-high SND words. If abstract concepts were simply less semantically rich than concrete concepts, one might expect that concrete (but not abstract) words would show effects of SND. Consistent with the results from the present study, Recchia and Jones (2012) found that a variable similar to SND was also able to significantly predict RTs in a lexical decision task. This finding was replicated in the current lexical decision data (Experiments 1 and 2), as well as extended within the context of other tasks requiring varying degrees of semantic processing (i.e., Experiment 3: progressive demasking task; Experiment 4: sentence relatedness task).

Most interestingly, we found that abstract words consistently produced effects of SND, whereas concrete words produced no effect (Experiments 1, 2, and 4) or a reduced effect (Experiment 3) of SND. This finding provides compelling evidence that linguistic associates are fundamental to abstract representations. In previous studies using concrete word stimuli (Pexman et al., 2008; Yap et al., 2011, 2012), facilitation effects for words associated with many physical features have been observed in a similar range of tasks as those used in the present study (e.g., lexical decision, progressive demasking, semantic classification), suggesting that sensorimotor properties may be central to concrete representations. This finding is consistent with the lack of SND effects for concrete words in our data.

### The Linguistic Complexity of Abstract Concepts

The assertion that linguistic associative information is more critical for abstract than concrete concepts is supported by several of the theories of lexical organization outlined earlier. For example, our conclusion is consistent with the theory of embodied abstract semantics (Vigliocco et al., 2009; Kousta et al., 2011), which states that linguistic associative information (of the type captured by SND) primarily underlies abstract representations, whereas sensorimotor information is more important for concrete representations. The different representational framework hypothesis (Crutch and Warrington, 2005) makes a similar argument regarding the abstract/concrete distinction in that it states that shared linguistic context (semantic association) is more important for abstract concepts, whereas concrete concepts are primarily organized by semantic similarity (i.e., same category, shared physical features)^7^. By virtue of the fact that SND captures large-scale co-occurrence patterns from human samples of language usage, it is able to reflect the semantic complexity of a concept beyond that which can be reflected based on sensorimotor properties alone. Therefore, we propose that the SND effects typically demonstrated by abstract (but not usually concrete) words in the present study are indicative of the greater semantic complexity of abstract words relative to concrete words. Although dual-coding theory is typically used to explain concreteness effects in word recognition (Paivio, 1971, 1991), the importance that this theory places on a verbal linguistic code for abstract words is also consistent with the present findings.

The proposed relative complexity of abstract representations is also supported by theoretical frameworks such as perceptual symbol systems (Barsalou, 1999; Barsalou and Wiemer-Hastings, 2005). Recall that this theory advocates for a common semantic system for concrete and abstract representations, given that both are activated by means of sensorimotor simulations. Although situational content is believed to be a feature of both word types, the situational content of concrete words primarily involves physically circumscribed objects within a specific context, whereas a diverse array of physical, introspective, and social events often characterizes abstract words. Given the extent of integration across content areas that would be necessary for a coherent abstract representation, it seems reasonable that widespread activation across various association areas would also be necessary at a neuroanatomical level to activate these words. Along these lines, adaptations of the hub-and-spoke model may explain the imaging findings of Pexman et al. (2007) (also see Moseley et al., 2013). For example, Binder and Desai (2011) propose that there are lower-level modal convergence zones (association areas) and higher-level convergence zones that store semantic representations in a hierarchical manner. Lower-level convergence zones are believed to store information about the sensorimotor features of concepts, whereas higher-level convergence zones bind information from lower level convergence zones to form supramodal representations. Although this view is similar to the hub-and-spoke model (Patterson et al., 2007; Lambon Ralph et al., 2010), Binder and Desai (2011) argue that there are several critical semantic hubs (throughout the lateral and ventral temporal cortex as well as the inferior parietal lobe) rather than a single semantic hub in the anterior temporal lobe. Consistent with this research, the findings from some recent neuroimaging investigations also suggest that abstract representations are neuroanatomically represented by widespread connections between an array of regions (e.g., Pexman et al., 2007; Moseley et al., 2013).

Support for the complexity of abstract concepts may also be illustrated by the nearest neighbors of concrete versus abstract words generated by WINDSORS. For example, the nearest neighbors for the concrete stimulus word *DEODORANT* are other concrete words with circumscribed meanings such as *SHAMPOO* and *AFTERSHAVE*. In contrast, the nearest neighbors for the abstract stimulus word *MASTERY* include other abstract words such as *SKILL* and *DEXTERITY*, whose meanings would conceivably require complex associations with a network of other concepts. The above-summarized neuroimaging findings are also consistent with the idea that abstract representations are typically acquired by generalizing across divergent examples illustrating a given concept (Moseley et al., 2013). For example, the meaning of the word *BRAVERY* may be represented by a combination of exemplars (e.g., a firefighter, someone battling cancer, a war veteran), all of which are associated with a wide variety of object-based and language-based features that contribute to the meaning of the abstract concept *BRAVERY.*

## Conclusion

Data from four different tasks that presumably vary in the extent to which they recruit semantic processing suggests that SND effects in visual recognition are robust. Moreover, SND appears to be especially sensitive to capturing the semantic complexities of abstract words. Finally, the current findings highlight the importance of examining interactive semantic effects, as these can reveal important insights into the underlying distinct semantic structures of various word types, including concrete and abstract concepts. As such, assumptions about visual word recognition based on studies only using concrete words should be challenged and examined using abstract words.

## Author Contributions

The experiments within this study were conducted as part of AD’s doctoral dissertation. AD was the primary author of this manuscript, and was responsible for statistical analysis of the data. LB was the faculty advisor and collaborator of this work. Both AD and LB contributed to the methodology and interpretation of findings.

## Conflict of Interest Statement

The authors declare that the research was conducted in the absence of any commercial or financial relationships that could be construed as a potential conflict of interest.

## Footnotes

1 The term “semantic distance” in the Buchanan et al. (2001) study is analogous to SND, except that these authors only statistically considered a given word’s 10 closest neighbors. Therefore, “low semantic distance” implied that neighbors were closely semantically related to the target, thus forming a dense neighborhood. In the same way, “high semantic distance” implied that neighbors were relatively distant from the target thus forming a sparse neighborhood. In contrast, in the present study the calculation of SND involved similarity (not distance) values. As such, high SND words have neighbors that are highly similar or closely semantically related to them (i.e., high SND words have low semantic distance to their neighbors). In the same way, low SND words have neighbors that are relatively less semantically related to them (i.e., have high semantic distance to their neighbors).

2 SND values theoretically range from 0 to 1, although the vast majority of words within the WINDSORS database have SND values under 0.5.

3 Levene’s test of equality of variances was significant for this comparison. As such, the degrees of freedom for the error term was adjusted accordingly.

4 As there was no rating available for the concrete – low SND stimulus word *STYROFOAM* in Kuperman et al.’s (2012) database, an average of the AoA ratings from the other concrete – low SND words was used as a proxy rating for this word.

5 Following Kousta et al.’s methodology, we collected these ratings using similar procedures as those used by Bradley and Lang (1999). However, instead of using the terms ‘unhappy’ and ‘happy’ as scale anchors, we used the terms ‘negative’ and ‘positive’ as scale anchors to refer to a range of possible positive and negative emotions that may be elicited for each word. In keeping with the range of scale values used by Bradley and Lang, participants in the present study provided a valence rating for each experimental word on a scale from 1(completely negative) to 9 (completely positive), with a rating of 5 representing a “neutral” rating.

6 In Experiment 3, the concreteness by SND interaction was only significant when subjects (but not items) were entered as a random effect.

7 The general framework of Crutch and Warrington’s (2005) concrete/abstract distinction is interpreted here as generally being consistent with our data, although specific claims of the Qualitatively Different Representational Hypothesis have not been replicated in several investigations (Hamilton and Coslett, 2008; Brozdowski et al., 2013; Geng and Schnur, 2015; Skipper-Kallal et al., 2015).
