# Supplementary material for: Semantic Neighborhood Effects for Abstract versus Concrete Words
Source: Front Psychol. 2016 Jul 6;7:1034. doi: 10.3389/fpsyg.2016.01034 (PMC4933712; doi:10.3389/fpsyg.2016.01034)
Supplement: Supplementary file 1 [file Table_1.DOCX]

Supplementary Material

Title: Semantic Neighborhood Effects for Abstract Versus Concrete Words

Authors: Danguecan, A.N., & Buchanan, L.

*** Correspondence:** Lori Buchanan: buchanan@uwindsor.ca

Supplementary Table 1

*Experimental Stimulus Words by Condition*

| Concrete – Low SND | Concrete – High SND | Abstract – Low SND | Abstract – High SND |
| --- | --- | --- | --- |
| Freezer | Booklet | Fervour | Discord |
| Woodpecker | Tablespoon | Concession | Banishment |
| Nostril | Tadpole | Acclaim | Penance |
| Subtitle | Flamingo | Infusion | Eviction |
| Crocodile | Gunpowder | Digestion | Cremation |
| Kangaroo | Mosquito | Cohesion | Fixation |
| Bayonet | Gorilla | Allergy | Vacancy |
| Volcano | Bazooka | Potency | Sorcery |
| Chandelier | Skyscraper | Absorption | Decryption |
| Aquarium | Ammonia | Fidelity | Nobility |
| Microphone | Microscope | Turbulence | Sustenance |
| Cutlery | Abdomen | Mastery | Modesty |
| Calculator | Embroidery | Saturation | Activation |
| Gymnasium | Incubator | Elevation | Asymmetry |
| Tablecloth | Chimpanzee | Conduction | Abstinence |
| Styrofoam | Intestine | Hydration | Excretion |
| Canister | Bungalow | Elegance | Accolade |
| Alligator | Deodorant | Adoration | Sterility |
| Pacifier | Cemetary | Sorority | Impurity |
| Container | Cigarette | Sensation | Deterrent |
| Prairie | Eardrum | Cuisine | Anguish |
| Lipstick | Necklace | Dampness | Prudence |
